# Lightweight Deep Learning Models for High-Precision Rice Seedling Segmentation from UAV-Based Multispectral Images

**DOI:** 10.34133/plantphenomics.0123

**Published:** 2023-11-30

**Authors:** Panli Zhang, Xiaobo Sun, Donghui Zhang, Yuechao Yang, Zhenhua Wang

**Affiliations:** ^1^ College of Engineering, Northeast Agricultural University, Harbin 150030, China.; ^2^ Aerospace Information Research Institute, Chinese Academy of Sciences, Beijing 100094, China.; ^3^ National Key Laboratory of Remote Sensing Information and Imagery Analyzing Technology, Beijing Research Institute of Uranium Geology, Beijing 100029, China.; ^4^ College of Agriculture, Northeast Agricultural University, Harbin 150030, China.

## Abstract

Accurate segmentation and detection of rice seedlings is essential for precision agriculture and high-yield cultivation. However, current methods suffer from high computational complexity and poor robustness to different rice varieties and densities. This article proposes 2 lightweight neural network architectures, LW-Segnet and LW-Unet, for high-precision rice seedling segmentation. The networks adopt an encoder–decoder structure with hybrid lightweight convolutions and spatial pyramid dilated convolutions, achieving accurate segmentation while reducing model parameters. Multispectral imagery acquired by unmanned aerial vehicle (UAV) was used to train and test the models covering 3 rice varieties and different planting densities. Experimental results demonstrate that the proposed LW-Segnet and LW-Unet models achieve higher F1-scores and intersection over union values for seedling detection and row segmentation across varieties, indicating improved segmentation accuracy. Furthermore, the models exhibit stable performance when handling different varieties and densities, showing strong robustness. In terms of efficiency, the networks have lower graphics processing unit memory usage, complexity, and parameters but faster inference speeds, reflecting higher computational efficiency. In particular, the fast speed of LW-Unet indicates potential for real-time applications. The study presents lightweight yet effective neural network architectures for agricultural tasks. By handling multiple rice varieties and densities with high accuracy, efficiency, and robustness, the models show promise for use in edge devices and UAVs to assist precision farming and crop management. The findings provide valuable insights into designing lightweight deep learning models to tackle complex agricultural problems.

## Graphical abstract

**Figure:**
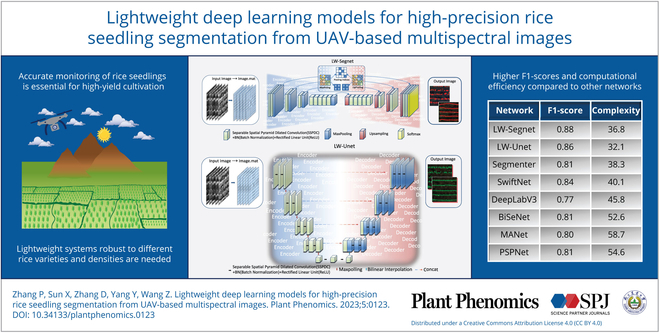

## Introduction

Accurately and precisely accessing field information is an important prerequisite for precision farming since it assists agricultural planning decisions and proper field management, which, in turn, contribute to profitability increase and cost reduction. Traditionally, the acquisition of crop information largely depends on manual efforts, which is time-consuming and labor-intensive for researchers conducting large-scale field measurements [1]. Thus, advanced remote sensing technique has become a popular method for obtaining the crop information in plant phenotyping, which mainly utilize the satellite-based platform [2], the ground-based platform [3], and the unmanned aerial vehicle (UAV)-based platform [4]. Generally, the costs of satellite-based systems are expensive and the spatial resolution is low; meanwhile, the accuracy is vulnerable to upper atmosphere, aerosols, and clouds. The ground-based systems always require cooperation between multiple sensors, which is complex and the portability is poor, not suitable for large-field monitoring processes. Of these, the use of UAV-based platform fills the gap of information between proximal ground sensing and meter spatial resolution platforms [5]. The UAV-based platform enables frequent aerial experiments as required, facilitating the acquisition of high-resolution spatial patterns. This approach captures substantial multi-temporal, high-definition imagery for effective crop monitoring.

The existing research on crop monitoring under the UAV-based platform has been covering the information like crop height [6], leaf area indexes [7], nitrogen [8], biomass, and yield estimation [9]. Overall, the approaches above have been extensively reported. However, because of the complex environment in rice transplanting field (e.g., paddy field and solar reflection in water field, which can easily cause imagery exposure), stand detection and plantation-row extraction in rice fields are still challenging and meaningful tasks, while benefiting precision farming in several applications, such as temporal analysis [10], navigation of agricultural robots [11], and the automatic driving of agricultural machinery [12]. The information could also be used to analyze the planting quality, predict productivity, and make important decisions during the management of the crop and for the next seasons. Furthermore, the access of crop rows’ information could help to evaluate the number of missed plants in each plantation row and, consequently, the amount of yield and the growth status [13]. Generally, the stand counts and row detection are most valuable at the initial stage of crop growth, for there are rare overlaps between the crops and the farmers still have time to re-transplant the field and make up the losses in time.

In general, the implementation of crop seedling row extraction is a method of line detection, and some traditional methods such as the Hough Transformation have been widely used to fit straight lines or known shape curves. Varela et al. [14] adopted the Canny Edge detector and Hough Transform method to detect the rows of plants. Su et al. [15] extracted the planted line of breeding corn in jointing stage by the Hough Transform method, while the extremum was found unstable with the growth of the crops, which made it difficult to detect the correct number of corn ridges. Varela et al. [5] implemented a workflow to identify plants in real field conditions and tested the UAV-based RGB imagery with a decision tree model, while the internal variance of non-corn objects should be reduced to improve the classification accuracy. As a matter of fact, although the traditional image processing algorithm could achieve qualified detection results, they assumed that the directions of crop rows were absolute horizontal and the lines were parallel, while the performance would be reduced as the lines were not straight and parallel in rice field, so that the ability of continuous plantation-rows’ tracking is still poor and exhibited poor robustness in a complex field environment. It is mainly because the traditional algorithms lack the capability to extract high-level semantic features of images, which leads to the poor accuracy of the model, and the performance is highly restricted by the image quality [16,17].

In recent years, deep learning (DL) methods have demonstrated much superior performance in many traditional computer vision applications including plantation rows and crop detection. Unlike traditional methods, seedling row extraction methods based on DL methods are general systems, and thus applicable across different types of crops. Pang et al. [18] proposed a DL method to process the entire row at one time instead of detecting individual crops in a row, eventually providing an emergence rate of the crop lines. However, the crop lines could be very long and the crop distribution is usually uneven in rice field, which may lead to a misjudgment of the seedling quality and is unfavorable for precision farming. Especially for plants like transplanting rice, 2 adjacent objects cannot be segregated properly; in the context of paddy rice stand and row detection, current methodologies exhibit limitations when applied to complex paddy field environments. This is particularly evident in the presence of light reflection and interference from the mud within the paddy fields. Wu et al. [19] reported the use of the regression network inspired by the deep fully convolutional neural network (CNN) to regress the density map and estimate the number of stands, and the results demonstrated a accuracy higher than 93% while the segmentation network appeared erroneously recognizethe non-plantation areas as rice areas. Kitano et al. [13] applied the DL method to measure the population of plants under different flight heights and plant densities, and they obtained the best results for the highest flight height under its specific Unet architecture, while the approach only present better results for lower plant densities that exhibited poor robustness. Miyoshi et al. [20] proposed a Multi-Stage Module based on a CNN architecture to detect single-tree species. However, the DL method they proposed exhibited poor robustness, which was only suitable for high-density object detection. Osco et al. [21] implemented a 2-branch architecture based on a CNN architecture and the results were superior to other deep networks, while the network’s architecture was too complex to reduce the amount of parameters, and it was almost impossible to integrate on the UAV platform to realize the real-time detection. Overall, the proposed DL models for stands and row detection exhibited poor robustness and low efficiency for plants with different density (high-density and low-density plantations). Normally, the failures of the network could be compensated by adding more complex network structures. However, this approach consumes a lot of computing resources, and requires a lot of manual annotation and iterative training in the early stage. For instance, the DeepLabv3+ model proposed by the University of Hong Kong [22] shows excellent performance on delineating the calving front, the application is limited by the large computational power (88 GB graphics processing unit [GPU] memory) and long training time (1 to 2 days), which limits the capability of edge computing.

To provide a more accurate decision-making process for precision agriculture on a UAV-based platform, the information access timeliness is crucial. Especially for the rice cultivars, where the decision window is brief, a rapid detection could help to mitigate or prevent yield decline with its production. Once the farming activities are delayed or advanced, it may lead to an opposite result [23]. In this regard, to the best of our knowledge, a lightweight DL architecture that is capable of counting plants and mapping plantation rows simultaneously for various rice cultivars under different planting densities is urgently needed in the field of farmland information detection. Consequently, in this study, we thus present highly robust and lightweight DL methods, which are the lightweight SegNet (LW-Segnet) model and the lightweight Unet (LW-Unet) model. Besides, these 2 models are compared with other state-of-the-art (SOTA) models. The precision and speed of the detection have been improved according to lightweight optimization. Our main contributions can be summarized as follows:

1. Introduction of 2 lightweight DL models: the LW-Segnet and LW-Unet

2. High precision and robustness of the models

3. Design of mixed lightweight convolution and spatial pyramid dilated convolution

4. Model performance on datasets of various rice varieties and densities

5. Computational efficiency of the models

A model possessing such capabilities offers an alternative to manual visual interpretation of crop fields, promoting sustainable agricultural system management. Certain crops, including citrus plants, corn, and numerous others, exhibit limited compensatory ability for vacant areas within a row, as they cannot occupy or lean towards these spaces. This constraint adversely affects yield per unit land area during the harvest season [24–27].

## Methods

### Study area

**Fig. 1.**
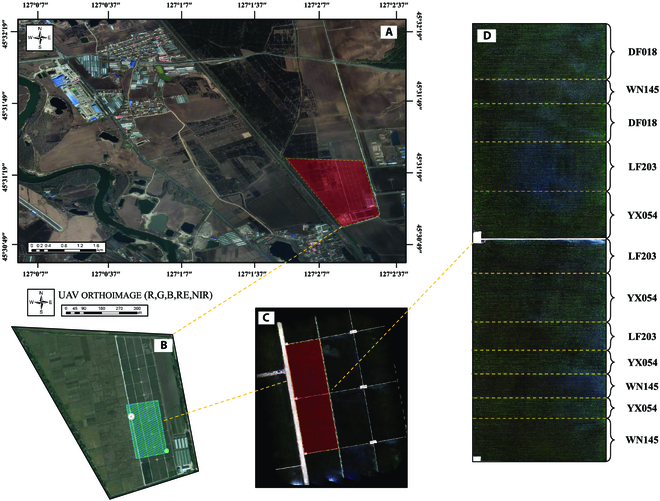
The Scientific Research Base for Whole Mechanization of Single Cropping Rice located in Northeast China. (A) Harbin City, Acheng District; (B) scientific research base; (C) imagery acquisition area; (D) region of interest.

The experiment was carried out at the Scientific Research Base for Whole Mechanization of Single Cropping Rice in North China (118°12′12″, 118°12′12″), Harbin City, Heilongjiang Province, which covers an area of 20 ha as illustrated in Fig. 1. The base is situated in a region characterized by a cold temperate continental monsoon climate, with an average annual temperature of 3.4 °C. The area experiences notable seasonal variations and warms up rapidly during the transplanting period. The annual rainfall typically ranges from 500 to 600 mm [28]. Dominant native rice cultivars are *Oryza sativa* L. in Northeast China, and the testing cultivars in the study area are YX054, LF203, and WN145. As suggested by the Standard Rules for high yield and rice production in Northeast China (Jiansanjiang Administration, 2015), all these cultivars were transplanted on 2021 April 15 by a transplanting machine with a plant spacing pattern of 300 mm × 150 mm (row spacing and plant spacing, respectively), and the planting area is 0.7 ha, 0.8 ha, and 0.7 ha for YX054, LF203, and WN145, respectively. The transplanting situation and characteristics of each cultivar are shown in Table 1.

**Table 1.** Transplanting situation and characteristics of the testing cultivars

Cultivars	Number of plants/ha	Plants per hole	Plants height (mm)
YX054	6.4×10^5^	5	133
LF203	9.6×10^5^	3	126
WN145	13.5×10^5^	2	115

### UAV platform and imagery acquisition

The UAV remote sensing data acquisition employed the DJI Phantom 4 multispectral flight (SZ DJI Technology Co., Shenzhen, China) for multispectral imagery acquisition in this study. The takeoff weight of the aircraft is 1,487 g, and the maximum ascent speed is 6 m/s. The camera sensors use six 1/2.9″ complementary metal-oxide-semiconductor, including 1 RGB sensor for visible light imaging and 5 monochrome sensors for multispectral imaging. Images for radiometric calibration of the camera were captured on the flat ground before and after each flight through a calibrated reflectance panel. The detailed information of the band wavelengths and the reflectance of the calibrated panel is listed in Table S1. Flights were carried out under clear and cloudless weather conditions between 11:00 a.m. and 11:30 a.m. The UAV traversed the trial area in fully automatic flight mode, configured using the DJI GS Pro software (https://www.dji.com/cn/groundstation-pro). The built-in real-time kinematic of the UAV could automatically obtain high-precision position and orientation system information (position parameters and attitude parameters when photographing) of the images for data georeferencing; about 3,400 frames of UAV-based multispectral images data were acquired in each flight with the forward and side image overlapping of 85% and 80%, respectively. To obtain images with a property resolution, the ground resolution was 30 mm for the images taken at the altitude of 70 m, 25 mm for the altitude of 50 m, and 20 mm for the altitude of 30 m. The weather was sunny without significant wind, so the possibility of image distortion due to weather conditions was eliminated.

Considering that the water vapor above paddy fields is more severe, the atmospheric correction is crucial for the inversion of real reflectance of transplanting rice in paddy field. The method of fast line-of-sight atmospheric analysis of spectral hypercubes (FLAASH) atmospheric correction model was used to eliminate the effects from oxygen, carbon dioxide, atmospheric molecular, and aerosol scattering, based on the moderate resolution transmission. The solar elevation and azimuth angle were read from a .BIL file, and the coordinates of the image center were 45°31′9.36″, 127°1′57.51″. The sensor altitude and ground altitude were 30 m and 151 m. The flight date was 2021 April 26, and the dates correspond to the transplanting stage of rice. The atmospheric model was set to mid-latitude winter, and the pix size was 0.016 m. The parameters were filled in FLAASH correction module in ENVI software. Obtaining radiance is a prerequisite to calculating the reflectance, and the radiance was calculated by Eq. 1.

L=gain×DN+bias (1)

where *L* represents the radiance, gain and bias represent the gain and deviation corresponding to sensor, and DN is the pixel gray value of the original image.

## Proposed Methods

### Related work

As a branch of machine learning, the DL method focuses on learning and obtaining more useful features from successive layers, while the core of DL lies in automatically learning from each layer, which is time-consuming [29]. The models proposed in these days tend to add more layers and deepen the structure of the model, and despite resulting in increased accuracy, they cannot meet the requirement of real-time and dynamic identification. Hence, the aim of the proposed method is to simplify the model structure, while improving the accuracy of segmentation. Before starting to propose a lightweight DL model, it is worthwhile to consider the time distribution on each layer. Therefore, a deep CNN architecture (ResNet), which ranked first during the ImageNet Large-Scale Visual Recognition Challenge [30], was adopted to analyze the computation time distribution on different layers. Figure S1A shows the computation time distribution with a batch size of 1 to 256, and it can be observed that the fully connected layer (FC) is the most time-consuming process when the batch size is small. However, a large batch size is usually chosen to train our dataset, which can help to accelerate the convergence speed and achieve the optimal accuracy. With the increase of the batch size, the most time-consuming layer turns out to be the convolution layers; the percentage of time reaches up to 92.10% when the batch size is 256, as shown in Figure S1B. Therefore, it is clear that a more efficient convolution layer should be designed and a modification of FC layers needs to be proposed to lighten the model without sacrificing the accuracy.

Row and stand detection can be regarded as a binary classification problem, where the pixels should be linked to a class label. For this goal, the semantic pixel-wise segmentation methods are proposed in our study. As a typical semantic segmentation model, the fully convolutional network (FCN) has been widely used in recent studies [31,32]. However, to lighten the convolution layers and the FC layers, the LW-Segnet and LW-Unet architecture, which consist of 2-stage encoder–decoder networks, are proposed in our study. Unlike FCN, the LW-Segnet and LW-Unet adopt the encoder–decoder structure. They contain an encoder that captures features from low level (e.g., rice seedlings) to high level (e.g., row lines) and a decoder that gradually recovers the spatial information. Therefore, they share an encoder that applies depthwise separable convolution to sense multiscale contextual information and a simple but effective decoder module to obtain sharp object boundaries [33]. In addition, The FC layers are discarded in 2 models; hence, it is only convolutional compared with the FCN. Overall, the LW-Segnet model and the LW-Unet model reduce the parameters of the model; therefore, the operation speed is increased. The architectures of the 2 models are shown in Figs. 4 and 5.

In the past, methods utilizing neural networks for crop seedling detection predominantly focused on classifying individual pixels. This entailed extracting feature vectors from a small region surrounding the target pixel and feeding them into the neural network. However, explicit modeling of the contextual relationships between adjacent pixels was lacking, hindering the extraction of global features from segmented image patches. In contrast, deep CNNs efficiently extract both global and local features. Throughout the entire CNN, images undergo successive convolutions, capturing contextual information between neighboring pixels via a series of convolutions, rather than a single preprocessing step for feature extraction. This approach facilitates the extraction of multilevel spatial and spectral features from the entire image and all its bands, thereby better capturing the multidimensional information within remote sensing imagery. Consequently, this study presents a novel lightweight deep CNN designed to extract multilevel spatial and spectral features from entire input images, facilitating information acquisition for crops in complex growth environments. To validate the superiority of our proposed method in terms of performance and efficiency, we compared it with several recent advanced techniques, including Segmenter: a Transformer-based image segmentation framework. Given the Transformer’s outstanding performance in natural language processing and computer vision tasks, as well as the popularity of Transformer architectures in generative pre-trained transformer models, we deemed Segmenter a significant point of comparison for this research [34]. SwiftNet is an efficient real-time semantic segmentation network that enhances performance through the employment of lightweight convolutional modules and effective upsampling and downsampling strategies. We selected SwiftNet as a comparison due to its focus on computational efficiency while maintaining accuracy [35]. DeepLabV3 is a semantic segmentation model built upon depthwise separable convolutions, utilizing atrous spatial pyramid pooling (ASPP) to enrich contextual information. As DeepLabV3 demonstrates significant performance improvements on large datasets, we included it in our comparison [36]. BiSeNet is a bilateral segmentation network that elevates segmentation performance by concurrently learning contextual information and high-resolution detail. We opted for BiSeNet as a comparison because it achieves high-precision segmentation with reduced computational complexity [37]. MANet is a multiscale attention network that captures context information across various scales through the use of multiscale attention modules. We incorporated MANet into our comparison due to its innovative approach in handling multiscale information [38]. PSPNet is a pyramid scene segmentation network that employs a pyramid pooling module to capture multiscale contextual information. We selected PSPNet for comparison because it exhibits robust performance in multiscale context modeling [39].

### The LW-Segnet network

As a 2-stage architecture, the LW-Segnet model contains both encoding and decoding structure. To make the model lighter and simpler, we have established an LW-SegNet architecture, which is composed of 4 encoders and 4 decoders. Each encoder layer corresponds to a decoder layer, and the final output of the decoder is sent to the softmax classifier to generate the probability for each pixel independently. Compared with the strategy by increasing the number of network layers, reasonable optimization of the network structure can better improve the segmentation effect [40]. In this paper, the proposed LW-SegNet model applies the depthwise separable convolution as the backbone instead of VGG16. The new structure not only reduces the number of parameters but also reduces the depth of the model, which makes the whole structure lighter and simpler.

### Lightweight hybrid attention mechanism module

To enhance the precision of rice seedling recognition and seedling strip extraction during the intricate transplanting stage, we incorporated a lightweight hybrid attention mechanism module prior to convolutional operations. This module comprises channel attention mechanisms for the 5 bands of multispectral imagery and spatial attention mechanisms within each channel. The primary function of the channel attention module is to adaptively allocate attention weights across different channels, bolstering the network’s perceptual capacity for diverse features. This is achieved by calculating each channel’s importance score and applying it to the feature maps, adjusting their weights to prioritize crucial channels, thereby elevating the quality of feature representation. To reduce computational overhead and parameter count, this study employs a lightweight channel attention mechanism designed with one-dimensional (1D) convolution, established in 3 steps:

1. Perform 1×1 max pooling and average pooling operations on single-band images for each of the 5 channels, obtaining two 1×5 pooling layers;

2. Utilize a 1×3 weight vector to conduct 1D convolution on max pooling and average pooling layers, yielding two 1×5 intermediate vectors;

3. Add the intermediate vector weights and pass them through a sigmoid function, obtaining a channel attention module within the 0 to 1 range.

Spatial attention mechanisms are techniques aimed at enhancing the performance of CNNs by adaptively allocating attention weights between different spatial locations, thus improving the network’s perceptual capacity for varying spatial positions. Spatial attention mechanisms calculate the importance scores for each spatial location and apply them to the feature maps, adjusting their weights to focus on essential spatial positions, in turn improving the quality of feature representation. The establishment of spatial attention mechanisms in this study can be divided into 3 steps:

1. Transform the original 5-channel imagery into 2-dimensional spatial vectors using max pooling and average pooling operations;

2. Add the 2-dimensional vectors and apply a 7×7 convolutional kernel for feature extraction, obtaining an intermediate weight vector;

3. Calculate a spatial attention module within the 0 to 1 range using a sigmoid function.

**Fig. 2.**
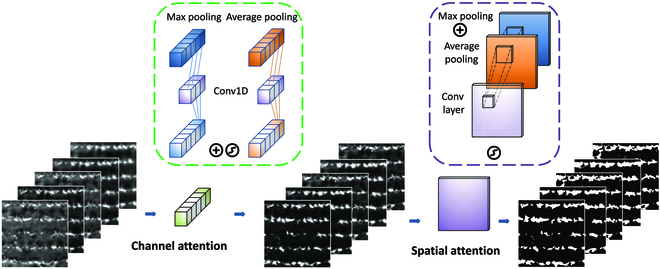
The lightweight hybrid attention (LHA) module.

The lightweight hybrid attention mechanism proposed in this study combines multiple attention mechanisms, offering remarkable advantages in processing multimodal data (Fig. 2). By adaptively selecting the most relevant attention mechanisms, this approach provides optimal modeling capabilities for each input modality, ultimately augmenting the neural model’s performance in complex tasks.

### Dilated spatial pyramid convolutions

To ensure feature extraction accuracy while reducing the parameter requirements of semantic segmentation models, this study introduces a dilated spatial pyramid convolution (DSPC) method for extracting seedling strip features and seedling information in complex paddy field environments. This method combines the advantages of dilated convolutions and pyramid convolutions, providing superior feature acquisition in intricate agricultural settings. Dilated convolutions maintain spatial resolution and expand the receptive field while reducing model parameters, ensuring precision in information extraction. Pyramid convolutions, on the other hand, more effectively handle multiscale information, preventing overfitting and enhancing model generalization capabilities. By improving the performance of convolutional networks, separable convolutions allow for parallel computation across different channels, accelerating convolution training speed. Specifically, DSPC performs convolution operations using multiple convolution kernels with varying dilation sizes and combines features of different scales through a pyramid structure to extract multiscale information. The convolution process is as follows:

1. Apply depthwise convolutions using dilated convolutions with sizes of 3×3, 5×5, and 7×7 on the 5-band imagery.

2. To increase the dimensions of the post-convolution imagery, use several 1×1×5 convolution kernels to perform pointwise convolutions on the output images after depthwise convolutions, deepening the dimensions of the output imagery.

### LW-Segnet model

**Fig. 3.**
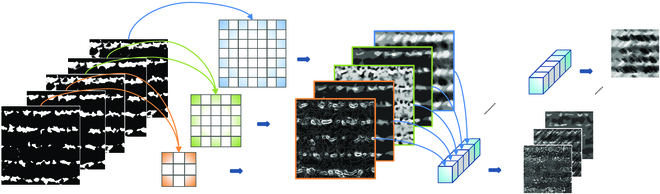
The process of separable spatial pyramid dilated convolution (SSPDC).

The LW-Segnet semantic segmentation model primarily consists of an encoding segment and a decoding segment, as illustrated in Fig. 3. The encoding layer employs a light hybrid attention-spatial pyramid dilated convolution (LHA-SSPDC) convolutional layer for feature extraction on the input image, followed by max-pooling for downsampling. The decoding layer serves to upsample the encoded feature image, yielding a segmentation result identical in size to the input image. The decoding layer similarly utilizes an LHA-SSPDC convolutional layer for feature extraction, followed by transposed convolution for upsampling, restoring the image’s original resolution. To facilitate addressing detail issues in semantic segmentation, SegNet employs pooling indices for skip connections. Skip connections are short connections from the encoding layer to the decoding layer, aiming to combine high-resolution feature maps from the encoding layer with low-resolution feature maps from the decoding layer for improved preservation of image detail information. Furthermore, the LW-SegNet model not only reduces the number of encoders and decoders but also optimizes the number of convolutional layers in between, significantly decreasing the model’s parameter count and complexity.

#### LW-Segnet encoding

**Fig. 4.**
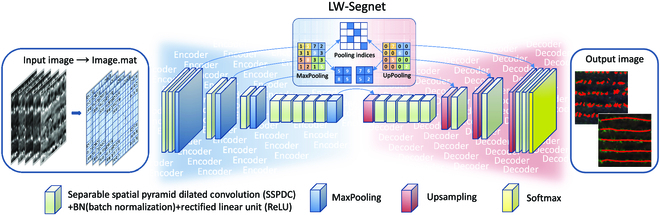
The overall structure of the LW-Segnet model.

The encoding process corresponding to the blue frame in Fig. 4 has 4 encoders. Firstly, the original 5-band images were converted into .mat format. These 4 encoders adopted the LHA-SSPDC instead of the original VGG16 to reduce the model size. The number of convolutions of each encoder varies, with 2, 1, 1, and 5 convolutions from the first encoder to the fourth encoder, respectively. After convolution, batch normalization was added to standardize the data distribution. Then, through the rectified linear unit (ReLU) layer, the network model can learn nonlinear relationships, improve model sparsity, speed up calculation, and avoid gradient disappearance. As the decoding layer deepens, the dimension of the feature images gradually increases to improve the feature extraction ability in complex environments. Meanwhile, max-pooling reduces the size of feature images, increases the field of view, improves the generalization ability of the model, and significantly reduces model parameters.

#### LW-Segnet decoding

The decoding process corresponding to the red frame in Fig. 4 adopts 4 decoders to achieve the purpose of lightweight simplification. The number of convolutions of each decoder also varies, with 5, 1, 1 and 2 convolutions from the first decoder to the fourth decoder, respectively. The corresponding decoders upsample their inputs using the received maximum pooling indices. The pooling indices produce sparse features instead of the deconvolution structure, which reduces the parameters while improving the edge scoring of the map. At the same time, the upsampling process does not involve parameter learning, which reduces the computational amount of the model. The encoder–decoder configuration of the LW-Segnet is shown in Table S2.

### The LW-Unet network

**Fig. 5.**
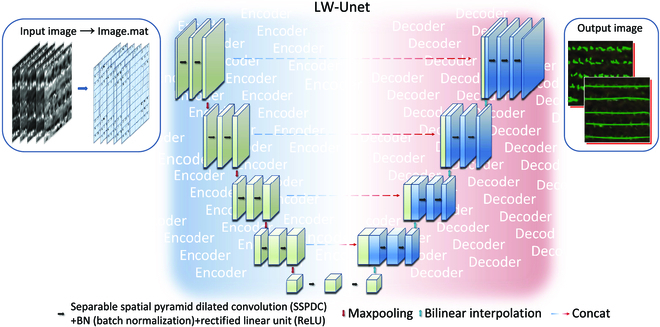
The overall structure of the LW-Unet model.

Previous studies applied the basic Unet model to solve the training tasks on small sample datasets, which achieved accurate results and demonstrated its feasibility on image segmentation [41,42]. However, the basic Unet model applies VGG16 as the backbone, which consumes more computing resources, resulting in large memory occupation. In particular, the convolution of multidimensional data could lead to exponential growth in parameters. Therefore, the LW-Unet that modified and expand in both encoding and decoding architecture of the basic Unet model was proposed in our study. The whole structure of the LW-Unet model is symmetrical, so that the original size of the image can be restored. The overall structure of LW-Unet model is shown in Fig. 5.

#### LW-Unet encoding

The LW-Unet encoding procedure corresponds to the blue frame depicted in Fig. 5. To reduce the model’s parameter complexity, the extraction process for the original 5-band image data transitions from standard convolution to LHA-SSPDC, aiming to diminish computational demands. Following each convolution, the addition of BN and ReLU layers normalizes and linearly activates the model, expediting convergence and enhancing computational efficiency. The decoding layer comprises 4 decoders, each with 2 convolution operations, and the intermediate layer’s feature image dimensions progressively increase to bolster feature extraction capabilities in complex environments. The implementation of max-pooling trims the output images of each decoder, augmenting the receptive field while simultaneously improving the model’s generalization capacity.

#### LW-Unet decoding

The LW-Unet decoding procedure corresponds to the red frame depicted in Fig. 5. Comprising 4 encoders, the input feature images for each encoder are assembled from 2 sources: one deriving from the output feature images of the corresponding decoder, and the other from upsampling operations. Channel-wise fusion occurs through concatenation of these 2 sets of features. In conventional Unet models, upsampling relies on transpose convolution; however, this study employs bilinear interpolation to reduce parameter complexity during upsampling. Consequently, intermediate layer images need not be dimensionally reduced, preserving feature image depth while diminishing model intricacy. Bilinear interpolation necessitates no parameter learning, thus decreasing computational demands and circumventing drawbacks associated with uneven overlap or checkerboard artifacts introduced by transpose convolution. Leveraging LHA-SSPDC, the concatenated feature images are continuously reduced in dimension and subsequently upsampled to restore the original size, resulting in the generation of the final, classified imagery. The encoder–decoder configuration of the LW-Unet is shown in Table S3.

### Network training

#### Dataset preparation

**Fig. 6.**
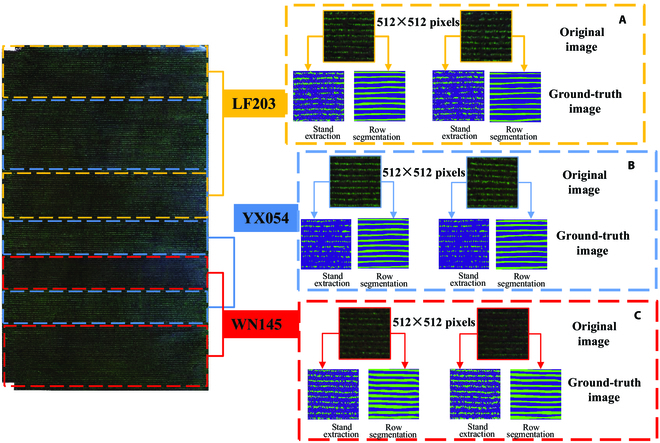
Data annotation and preprocessing. (A) The original image and ground-truth image of LF203. (B) The original image and ground-truth image of YX054. (C) The original image and ground-truth image of WN145.

Through the acquisition of imagery depicting rice seedlings at the transplanting stage, the training data sample encompasses a remote sensing image of 3 rice cultivars—LF203, YX054, and WN145—at this stage, boasting a resolution of 8,076 × 27,058 pixels. The entire field adhered to a randomized complete block design, wherein each cultivar was allotted to a random block. Notably, YX054 featured sparse planting, low density, and larger plants, while WN145 exhibited smaller plants and higher planting density. LF203, on the other hand, maintained a moderate planting interval. Utilizing Labelme, an annotation software for segmentation tasks, data annotation was completed. Directly inputting the entire image risks overwhelms the RAM, and the proposed LW-Segnet and LW-Unet models necessitate 512×512 pixel input images. Consequently, the original remote sensing image was subdivided into 1,300 images at the aforementioned resolution. The dataset was apportioned in a 6:1:3 ratio, corresponding to the training, validation, and testing sets, as delineated in Table 2. Concurrently, data preprocessing was executed, encompassing (a) remote sensing orthophoto image mosaicking, (b) cropping to the area of interest, and (c) image augmentation, such as reversing, rotating, and resampling. To ensure seamless rice seedling rows within predicted images, the segmented imagery’s seedling ends were manually smoothed and connected, establishing the ground truth, as illustrated in Fig. 6.

**Table 2.** Distribution of data across training, validation, and testing sets

Dataset	Images (512×512)		
	Train 60%	Validation 10%	Test 30%
YX054	2,280	380	1,140
LF203	1,980	330	990
WN145	1,740	290	870

#### Loss function and optimizer settings

The selection of an appropriate loss function is crucial in evaluating the discrepancy between predicted and true labels. The cross-entropy loss function, also referred to as logarithmic or logistic loss, is commonly employed in the majority of semantic segmentation contexts [43], demonstrating efficacy in pixel-level classification. Primarily utilized for binary pixel-level classification. However, it exhibits a notable shortcoming: when tasked with segmenting foreground and background, if the quantity of foreground pixels is significantly smaller than that of background pixels, the background component within the loss function will dominate, resulting in a model heavily biased toward the background and subpar performance [44]. The cross-entropy loss function can be defined as per Eq. 2:

$$L_{\mathrm{BCE}}\left(y,p\right)=-\frac{1}{n}\sum_{1}^{n}\left(y\cdot \text{log}\left(p\right)+\left(1-y\right)\cdot \text{log}\left(1-p\right)\right)$$ (2)

where *y* represents the ground truth, *p* denotes the predicted value, and *n* signifies the quantity of data within the batch.

Consequently, the weight binary cross-entropy (WBCE) loss was chosen to train the models proposed in our investigation. Considering that the positive samples (seedling rows) constitute a minute fraction relative to negative samples (background), we introduced α and γ to enhance seedling row extraction. The role of α is to weight the loss of different sample classes, amplifying the weight of positive sample loss when positive samples are scarce. Conversely, γ serves to direct the network’s focus toward the optimization of challenging tasks when hard training samples are abundant and easy samples are scarce. This approach bolsters the model’s recall rate and mitigates the issue of data imbalance to a certain degree.

$$\begin{array}{c} L_{\text{WBCE}}\left(y,p\right)=\\ -\frac{1}{n}\sum_{1}^{n}\left(\alpha {\left(1-p\right)}^{\gamma }\cdot y\text{log}\left(p\right)+\left(1-\alpha \right)p^{\gamma }\left(1-y\right)\cdot \text{log}\left(1-p\right)\right) \end{array}$$ (3)

where γ is the focusing parameter that smoothly adjusts the rate at which easy examples are downweighted. α is the balancing parameter, which is 0.25. According to the recommendation in [45], we set γ = 2 and the balancing parameter α = 0.25.

The Adam optimizer is used to train 50 rounds. The initial learning was set to 0.0001, and the batch size was set to 32. The optimal learning rate and batch size are determined through multiple experiments [40].

#### Tasks and metrics

The objective of our model is to parse the scene and segment the rice seedling with high, medium, and low planting density, which was based on the remote sensing data taken by the multispectral UAV. The precision (*P*), recall (*R*), F1-score (*F*1), mean intersection of union (*mIoU*), and overall accuracy (*A*) metrics are used for the fair evaluation of the segment results, which are defined as follows:

$$\text{Precision}\;\left(P\right)=\frac{\mathrm{TP}}{\left(\mathrm{TP}+\mathrm{FP}\right)}$$ (4)

$$\text{Recall}\;\left(R\right)=\frac{\mathrm{TP}}{\left(\mathrm{TP}+\mathrm{FN}\right)}$$ (5)

$$\text{Intersection over Union}\;\left(\mathrm{IoU}\right)=\frac{\mathrm{TP}}{\left(\mathrm{TP}+\mathrm{FP}+\mathrm{FN}\right)}$$ (6)

$$F1-\text{score}\;\left(F\right)=\frac{2\mathrm{PR}}{\left(R+P\right)}=\frac{2\mathrm{TP}}{\left(2\mathrm{TP}+\mathrm{FP}+\mathrm{FN}\right)}$$ (7)

where True Positive (TP) is the count of pixels that are true positives (pixels correctly segmented as the seedling and seedling rows), True Negative (TN) is the count of pixels that are true negatives (pixels correctly segmented as the non-seedling and non-seedling rows), False Positive (FP) is the count of pixels that are false positives (pixels falsely segmented as the seedling and seedling rows), and False Negative (FN) is the count of pixels that are false negatives (pixels falsely detected as the non-seedling and non-seedling rows).

To segment the rice seedling rows with highly imbalanced targets and background, the FN needs to be minimized and therefore ensure the percentage of correctly predicted seedling rows, so the value of recall is more crucial for row segmentation than precision in our study [46]. Besides, the precision and recall are mutually constrained to one another; a model with high precision always leads to a low recall, and vice versa. This contradictory nature leads us to consider these 2 metrics together. Therefore, we introduced the F1-score to comprehensively assess the 2 metrics, recall and precision, which will be higher when high precision and high recall are achieved simultaneously [47], and also shows a higher quality of the model.

#### System setup

To ascertain the segmentation accuracy and computational time of the 2 proposed networks, an examination of seedling row detection and stand count is conducted. The experiment is executed on a Windows 10 operating system, utilizing a desktop equipped with an AMD Ryzen 7 5800X 8-Core Processor @ 3.80 GHz and 128 GB RAM. Python serves as the programming language, with PyCharm as the compiler. Furthermore, network model construction, training, and testing are accomplished via the Keras DL library and the TensorFlow 1.0 DL framework. An NVIDIA GeForce GTX 2080 with 16 GB of memory facilitates network training during the experimentation process.

## Results and Discussion

### Comparison of network efficiency

A primary objective of this study is to explore the comprehensive capabilities of semantic segmentation models in crop segmentation, necessitating a balance between expressiveness and computational efficiency across various model metrics—specifically, an analysis of GPU memory usage, model complexity, parameter quantity, and execution speed. Six recently proposed lightweight semantic segmentation models are compared to the LW-Segnet and LW-Unet models presented in this study, with comparison results tabulated in Table 3. As semantic segmentation model complexity increases, so does performance, albeit at the cost of greater computational demands and slower training and inference processes. Evidently, the proposed LW-Unet model exhibits the lowest complexity, followed by the LW-Segnet model, while the MANet model boasts the highest complexity, increasing by 82.9% compared to the LW-Unet model.

**Table 3.** Quantitative comparison with state-of-the-art (SOTA) lightweight networks

Network	GPU memory (MB)	Complexity (GFLOPs)	Parameters (million)	Speed (FPS)
Segmenter [34]	742.6	38.3	9.2	105.3
SwiftNet [35]	600.2	40.1	13.4	85.2
DeepLabV3 [36]	624.7	45.8	12.7	80.3
BiSeNet [37]	960.2	52.6	15.3	88.1
MANet [38]	1,014.5	58.7	16.8	65.3
PSPNet [39]	572.1	54.6	14.6	73.7
LW-Segnet	693.4	36.8	11.0	117.5
LW-Unet	603.7	32.1	10.6	143.3

Real-time semantic segmentation systems require models with sufficiently rapid inference speeds to accommodate high frame rates per second. Among the 6 network structures, the LW-Unet model boasts the fastest inference speed, followed by the LW-Segnet model, while the MANet model’s inference speed is the slowest, decreasing by 54.4% compared to the LW-Unet model. Consequently, network complexity and model inference speed exhibit a negative correlation, with the LW-Segnet model demonstrating the lowest complexity and fastest inference speed among the 6 networks.

Semantic segmentation models typically employ large input images, and an excessive parameter count may easily result in GPU memory overflow. Consequently, model design must consider GPU memory consumption, ensuring that parameter quantity and intermediate feature maps do not exceed GPU memory limitations. The PSPNet model consumes the least GPU memory during training, signifying a relatively lower quantity of model parameters and intermediate results stored in GPU memory—performance in this regard is similarly commendable for the LW-Unet, SwiftNet, and Deep LabV3 models. Greater parameter quantities generally indicate a more robust representational capacity, although this may also entail increased storage and computational demands and a heightened risk of overfitting.

Among the 6 lightweight network structures, model parameter quantities are consistently low, with the Segmenter model boasting the fewest parameters, followed by the LW-Unet model; the MANet model, conversely, exhibits the highest parameter count, surpassing the Segmenter model by 66.3%. Upon comprehensive comparison of the efficiency of the 6 network models, the LW-Segnet and LW-Unet models demonstrate excellent overall performance, characterized by lower parameter quantities, GPU memory usage, and complexity, as well as faster inference speeds.

### Stand extraction of 3 rice cultivars

To holistically evaluate the merits and shortcomings of the proposed LW-Segnet and LW-Unet models compared to 6 SOTA networks in stand extraction, the calculated precision, recall, IoU, and F1-score values for the extraction results of YX054, LF203, and WN145 rice seedling varieties are presented in Tables 4, 5, and 6, respectively. For the YX054 variety, the SwiftNet network achieves the highest precision and lowest recall for rice seedling row extraction, while the DeepLabV3 network exhibits the lowest precision and highest recall. This discrepancy primarily stems from the limited range of DL networks supported by SwiftNet compared to mainstream frameworks such as TensorFlow and PyTorch, which results in weaker extraction capabilities for intricate features in rice seedling row extraction outcomes, leading to a lower recall. The high precision of SwiftNet can be attributed to the extensive background pixels of rice seedlings.

**Table 4.** Quantitative comparison results of the stand extraction on the YX054 test set with the SOTA networks. Bold text represents the optimal values within the SOTA domain.

Network	Precision (%)	Recall (%)	IoU (%)	F1-score
Segmenter	78.25	84.10	70.20	0.81
SwiftNet	**92.64**	76.32	78.32	0.84
DeepLabV3	67.33	**90.32**	64.43	0.77
BiSeNet	73.52	89.63	66.82	0.81
MANet	77.63	82.72	72.36	0.80
PSPNet	75.62	86.21	68.78	0.81
LW-Segnet	91.23	85.15	**83.42**	**0.88**
LW-Unet	90.12	82.31	80.61	0.86

**Table 5.** Quantitative comparison results of the stand extraction on the LF203 test set with the SOTA networks. Bold text represents the optimal values within the SOTA domain.

Network	Precision (%)	Recall (%)	IoU (%)	F1-score
Segmenter	81.03	83.27	70.73	0.82
SwiftNet	**95.41**	75.02	72.50	0.84
DeepLabV3	84.66	77.41	79.76	0.81
BiSeNet	79.53	79.24	74.06	0.79
MANet	73.24	87.48	65.26	0.80
PSPNet	70.76	**91.20**	68.34	0.80
LW-Segnet	90.10	84.28	**85.45**	**0.87**
LW-Unet	92.32	80.25	82.39	0.86

**Table 6.** Quantitative comparison results of the stand extraction on the WN145 test set with the SOTA networks. Bold text represents the optimal values within the SOTA domain.

Network	Precision (%)	Recall (%)	IoU (%)	F1-score
Segmenter	84.42	76.10	74.37	0.80
SwiftNet	81.38	79.17	78.21	0.80
DeepLabV3	91.08	88.83	80.14	0.90
BiSeNet	71.24	89.72	65.42	0.79
MANet	74.50	86.48	68.33	0.80
PSPNet	78.33	82.04	72.40	0.80
LW-Segnet	**93.34**	**90.25**	**87.06**	**0.92**
LW-Unet	87.92	85.42	83.28	0.87

**Fig. 7.**
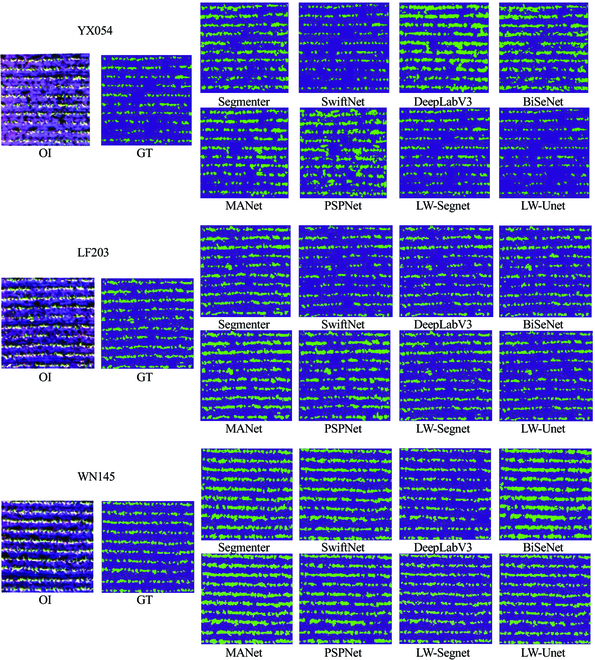
Examples of stand detection in YX054, LF203, and WN145 datasets. Each image is 512×512 pixels. From top to bottom, the examples are from scenes of YX054, LF203, and WN145 seedlings, respectively. From left to right are the visualization results of original images (OI), the ground truth (GT), and the results of Segmenter, SwiftNet, DeepLabV3, BiSeNet, MANet, PSPNet, LW-Segnet, and LW-Unet, respectively.

The DeepLabV3 network structure can divide image patches into boundary and interior pixels, processing them separately to enhance segmentation accuracy at the boundaries. This approach allows the model to extract more features without sacrificing spatial resolution, yielding more detailed segmentation results. However, DeepLabV3 primarily relies on contextual information for segmentation and is insufficient in utilizing local detail information, which affects the segmentation of small targets or intricate features, resulting in lower precision. The classification results of the 2 network structures are illustrated in Fig. 7.

The F1-score serves as a comprehensive metric for evaluating model accuracy, encompassing both precision and recall of the classification model. As the results show, the LW-Segnet model achieves the highest F1-score, demonstrating optimal performance in extracting YX054 rice seedling variety features. Furthermore, the LW-Segnet model’s IoU values consistently surpass those of other SOTA models, indicating that the predictions generated by the LW-Segnet model are more accurate in capturing features specific to the YX054 rice seedling variety.

For the LF203 variety, the SwiftNet network similarly achieves the highest precision and lowest recall values in rice seedling row extraction results, while the PSPNet network exhibits the lowest precision and highest recall values. Remarkably, PSPNet can attain excellent boundary accuracy without the need for additional boundary detection branches, which indicates that the pyramid pooling module embedded within PSPNet is well-suited for semantic segmentation. By effectively utilizing context information from different scales through the pyramid pooling module, PSPNet may cause internal target pixels to be misclassified as background, consequently reducing precision. Furthermore, PSPNet lacks a dedicated boundary detection branch and solely relies on the output from the pyramid pooling module for segmentation, leading to higher probabilities of misclassification at target boundaries and diminished precision.

As evidenced by Table 5, the LW-Segnet model achieves the highest F1-score for the extraction of LF203 seedlings, whereas the BiSeNet model scores the lowest. BiSeNet employs lightweight dilated convolutions to construct the context path, allowing for an expanded field of view without significant loss of spatial information, which is beneficial for enhancing segmentation performance. However, the limited spatial perception capabilities of BiSeNet’s spatial detail path cannot fully rectify the misclassifications generated by the context path, consequently reducing precision.

Among the 8 SOTA models, MANet has the lowest IoU value for the extraction of LF203 rice seedlings. By leveraging the attention mechanism, MANet can automatically learn key information from feature maps, effectively enhancing the model’s feature representation capabilities and achieving high segmentation performance. Nonetheless, the overall network structure of MANet is relatively simplistic, and its modeling capacity for complex scenarios and edge regions is limited, resulting in greater information loss and higher misclassification rates. Similarly, the LW-Segnet model demonstrates optimal performance in IoU values for the extraction of LF203 rice seedlings.

For the WN145 variety, the LW-Segnet network achieves the highest precision, recall, IoU, and F1-score in rice seedling row extraction results. On the other hand, BiSeNet exhibits the poorest precision and lowest IoU values for seedling row extraction, as well as the lowest F1-score. This may be due to BiSeNet’s reliance on 2 auxiliary losses (edge loss and hole loss) to constrain segmentation results, but merely using loss function constraints proves insufficient. While these 2 losses can prevent some edge information loss and small region under-segmentation, their overall impact on improving accuracy remains limited. Moreover, BiSeNet employs ResNet18 as its backbone and removes the max-pooling layer, allowing for the capture of richer spatial details. However, ResNet18’s shallow depth yields limited semantic extraction capabilities, contributing minimally to accuracy improvement.

Crucially, BiSeNet’s training primarily targets pixel accuracy, prompting the model to prioritize recall enhancement while neglecting precision changes, as illustrated in Table 6. The Segmenter network structure exhibits lower recall and poorer F1-score values for the extraction of WN145 seedlings, which may be attributed to its lack of an attention module, similar to BiSeNet. This deficiency weakens the model’s focus on key areas, hindering the learning of precise segmentation strategies and making it difficult to enhance accuracy. Furthermore, Segmenter’s training objective solely concerns pixel accuracy, overlooking changes in precision. Relying on pixel accuracy alone as a loss function proves challenging for promoting model improvement in precision.

In summary, when extracting WN145 rice seedling variety, the LW-Segnet network model demonstrates optimal performance, followed by the DeepLabV3 network model, while the BiSeNet network model fares the worst.

Although different DL semantic segmentation network models exhibit varying segmentation effects on the same rice variety, the performance of a single semantic segmentation model also differs when applied to 3 rice varieties with varying seedling sizes. When segmenting smaller rice seedlings of the YX054 variety, DeepLabV3 demonstrates extremely low precision, but it presents superior precision and recall values for larger, denser varieties LF203 and WN145. This indicates that DeepLabV3 possesses relatively poor robustness, and its cross-entropy loss function is more suitable for high-density target segmentation [48]. Cross-entropy loss achieves better classification for high-density targets, as each pixel is surrounded by similar pixels. However, for low-density targets, neighboring pixels may belong to different classes, rendering cross-entropy loss less applicable and necessitating the consideration of alternative losses, such as dice loss [49].

Furthermore, DeepLabV3’s feature pyramid structure is not entirely suitable for large-scale contextual encoding. Employing a larger receptive field and longer connections to capture richer contextual information could enhance segmentation performance for low-density targets. Similarly, the BiSeNet network structure exhibits significant performance differences in seedling row extraction for different rice varieties. In the extraction experiments for smaller YX054 seedlings and larger, denser WN145 seedlings, the BiSeNet network structure demonstrates weaker extraction capabilities and lower F1-score values. However, it performs better in segmenting moderately sized LF203 seedlings, mainly due to BiSeNet’s utilization of upsampling and downsampling structures. These structures enable better multiscale feature fusion for targets of intermediate density, but they lose more information during the process for targets with higher or lower density, resulting in inferior feature representation capabilities. Additionally, BiSeNet’s contextual information is relatively limited. Its context extraction module primarily relies on dilated convolutions, which possess a smaller receptive field and weaker modeling of larger contextual ranges. This impacts the segmentation of low-density and high-density targets, which require richer contextual information.

Among the 8 SOTA models, the LW-Segnet network structure consistently achieves optimal segmentation performance for rice seedlings of different densities. This highlights the robustness of the LW-Segnet network model in seedling row extraction, primarily due to the encoder–decoder structure that caters to both intricate and redundant feature extraction. The proposed lightweight hybrid attention mechanism module adaptively allocates attention weights across different channels, enhancing the network’s perception of various features. By integrating the advantages of dilated convolutions and pyramid convolutions, the model better handles feature acquisition in complex agricultural environments. Dilated convolutions maintain spatial resolution and increase receptive fields while reducing model parameters, ensuring information extraction accuracy. Concurrently, the pyramid structure combines features of different scales to extract multiscale information.

### Seedling row extraction of 3 rice cultivars

**Fig. 8.**
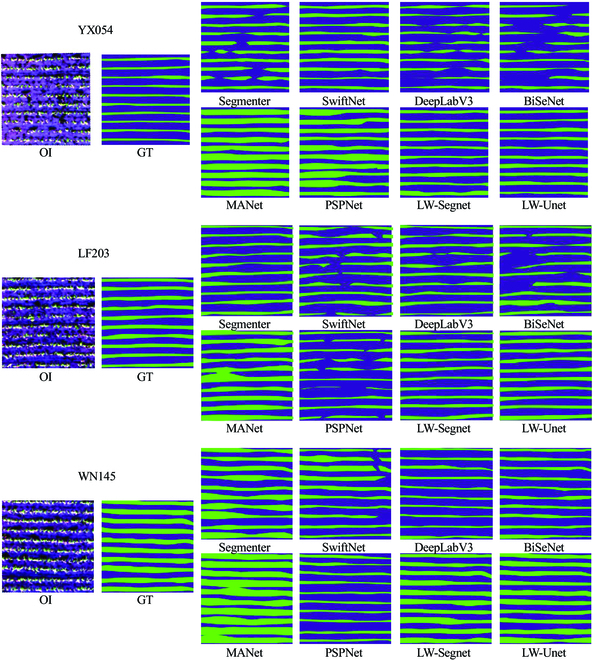
Examples of seedling row extraction in YX054, LF203, and WN145 datasets. Each image is 512×512 pixels. From top to bottom, the examples are from scenes of YX054, LF203, and WN145 seedlings, respectively. From left to right are the visualization results of OI, the GT, and the results of Segmenter, SwiftNet, DeepLabV3, BiSeNet, MANet, PSPNet, LW-Segnet, and LW-Unet, respectively.

To comprehensively evaluate the merits and demerits of the proposed LW-Segnet and LW-Unet models in comparison to 6 SOTA networks for crop seedling feature extraction, segmentation results of evaluation metrics are presented in Tables 7, 8, and 9. As can be gleaned from the tables, for the YX054 variety, the DeepLabV3 network achieves the highest precision in rice seedling feature extraction. This is primarily due to its ability to effectively extract only a portion of the ground truth image, resulting in a higher proportion of correct predictions in the seedling feature extraction results, as illustrated in Fig. 8. However, the DeepLabV3 network exhibits suboptimal completeness in seedling feature extraction. Utilizing a relatively small dilated convolution kernel (3×3), it can extract spatial details effectively, contributing to enhanced precision but with a limited receptive field, making it challenging to capture global context and affecting recall, which is the primary reason for its inferior recall performance.

**Table 7.** Quantitative comparison results of the seedling row extraction on the YX054 test set with the SOTA networks. Bold text represents the optimal values within the SOTA domain.

Network	Precision (%)	Recall (%)	IoU (%)	F1-score
Segmenter	73.25	68.82	68.46	0.71
SwiftNet	77.20	72.28	79.56	0.75
DeepLabV3	**88.41**	63.21	76.10	0.74
BiSeNet	82.25	59.70	72.25	0.69
MANet	54.31	**90.45**	50.77	0.68
PSPNet	60.03	88.26	54.21	0.71
LW-Segnet	80.24	85.52	81.63	0.83
LW-Unet	86.43	87.61	**84.28**	**0.87**

**Table 8.** Quantitative comparison results of the seedling row extraction on the LF203 test set with the SOTA networks. Bold text represents the optimal values within the SOTA domain.

Network	Precision (%)	Recall (%)	IoU (%)	F1-score
Segmenter	83.28	71.85	76.41	0.77
SwiftNet	70.07	68.83	68.42	0.69
DeepLabV3	80.32	75.82	72.23	0.78
BiSeNet	72.01	63.54	64.20	0.68
MANet	58.84	**85.41**	59.88	0.70
PSPNet	84.29	50.02	50.13	0.63
LW-Segnet	81.25	80.60	80.24	0.81
LW-Unet	**87.65**	83.23	**86.43**	**0.86**

**Table 9.** Quantitative comparison results of the seedling row extraction on the WN145 test set with the SOTA networks. Bold text represents the optimal values within the SOTA domain.

Network	Precision (%)	Recall (%)	IoU (%)	F1-score
Segmenter	78.82	69.82	73.03	0.74
SwiftNet	73.21	74.48	69.41	0.74
DeepLabV3	90.01	83.01	56.32	0.86
BiSeNet	87.65	81.05	60.34	0.84
MANet	61.04	85.01	61.29	0.71
PSPNet	**91.92**	50.23	51.30	0.65
LW-Segnet	82.17	80.01	84.41	0.81
LW-Unet	89.92	**87.72**	**87.42**	**0.89**

The BiSeNet network model has the lowest recall, mainly due to its simple feature fusion approach (concatenation and 1×1 convolution), which fails to effectively integrate the feature information of high- and low-resolution sub-networks, making it difficult to achieve complementary information, which is the primary reason for its poor recall. The results of the YX054 variety seedling information extraction indicate that the MANet model achieves the highest recall but with extremely low precision. This is primarily attributed to the adoption of dense connection designs, multi-path structures, transition layers with skip connections, attention mechanisms, ASPP modules, and the retention of all spatial resolutions. These designs maximize image information learning and enhance the model’s perceptual capabilities. The heightened perceptual abilities increase the likelihood of overfitting, leading to higher recall but lower accuracy in prediction. However, overall, the LW-Unet model demonstrates the most optimal IoU value and the highest F1-score, indicating that its predictions for extracting YX054 variety rice seedling features are more accurate.

For the seedling row extraction results of the LF203 rice variety, the MANet network structure’s extraction accuracy remains the poorest, while the extraction accuracy of the LW-Unet network model proposed in this study is the highest. Among the 8 SOTA models, the LW-Unet model’s comprehensive extraction capability for the LF203 rice variety seedlings remains the strongest. Conversely, the PSPNet network structure exhibits the lowest recall, IoU value, and F1-score for seedling feature extraction, resulting in the poorest segmentation accuracy. This is due to PSPNet’s reliance on lower-level features as input for segmentation, rendering it less capable of accurately extracting intricate image features, which significantly impacts segmentation accuracy. Furthermore, after feature pyramid pooling, PSPNet merely employs simple concatenation to fuse features of different scales, preventing effective feature complementation and enhancement, which affects the final segmentation accuracy. Additionally, the absence of modules that improve the model’s perceptual capabilities, such as attention mechanisms and residual connections, also contributes to the diminished segmentation accuracy of PSPNet.

For the WN145 variety, the LW-Unet network achieves the highest recall, IoU, and F1-score in rice seedling row extraction results. Conversely, the MANet model exhibits the poorest precision in seedling row extraction, while the PSPNet network displays the lowest recall, IoU, and F1-score values. However, both the DeepLabV3 and BiSeNet network models demonstrate excellent performance in rice seedling row extraction outcomes. This is primarily due to their utilization of high-level semantic features as input for segmentation, which enables the capture of advanced semantic information and enhances the models’ comprehension of complex targets, thereby improving IoU and F1-score values. Additionally, both DeepLabV3 and BiSeNet employ cross-entropy loss functions and prioritize optimization for smaller targets, which further bolsters the models’ learning capabilities for small objectives and subsequently boosts IoU and F1-score values. In summary, DeepLabV3 and BiSeNet, by employing higher-level semantic features, robust feature fusion mechanisms, moderate downsampling strategies, intricate network structures, and loss optimization for small targets, exhibit strong feature representation and global understanding capabilities. This allows them to effectively learn intricate features of complex targets, such as rice seedlings, resulting in superior performance in IoU and F1-score metrics.

Unlike the experimental results exhibited in rice seedling row extraction, the 8 SOTA models display varying yet relatively consistent effects in rice seedling belt extraction trials across different rice varieties. Among the 8 SOTA models, MANet and PSPNet demonstrate poorer extraction performance for seedling belt information. PSPNet, in particular, is more prone to overfitting when extracting low-density rice seedling belts. This is due to its use of a simplistic feature pyramid pooling module, which cannot effectively fuse semantic information of different scales, resulting in weaker expressive and generalization capabilities, and thus a higher tendency for overfitting during low-density information extraction. Conversely, the LW-Unet model achieves the best seedling belt extraction performance.

SwiftNet, DeepLabV3, and BiSeNet models tend to generate discontinuous seedling belts during extraction, leading to lower IoU values, as depicted in Fig. 8. SwiftNet employs an overly simplistic feature extraction module that fails to effectively fuse multiscale semantic information, resulting in weaker global understanding and boundary localization capabilities, which contribute to the lower IoU values. Although DeepLabV3 and BiSeNet utilize ASPP modules and twin network structures to merge multiscale information, the feature fusion remains suboptimal, with both types of information struggling to fully complement each other, thereby affecting IoU values. Additionally, these 3 network structures employ high-level input features, which, while providing rich semantic information, have lower spatial resolution, making it challenging to accurately pinpoint target boundaries. Furthermore, these structures have higher downsampling rates, causing significant loss of spatial information. Although resolution is eventually restored through upsampling, it is difficult to fully recover fine-grained details, which also contributes to lower IoU values.

Among the 8 SOTA models, the LW-Unet network structure consistently achieves optimal segmentation results when extracting seedling belts of varying densities, demonstrating a high degree of robustness. This is primarily attributed to the proposed LHA-SSPDC convolution, which enhances the network’s perceptual capabilities for different features while satisfying lightweight requirements. Additionally, the model employs bilinear interpolation for upsampling, negating the need for dimensionality reduction in intermediate layer images, thereby preserving the depth information of feature images and improving the model’s accuracy.

## Conclusion

This study presented 2 lightweight neural network architectures, which is the LW-Segnet and LW-Unet for high-precision rice seedling segmentation utilizing multispectral UAV imagery. The lightweight designs consisting of hybrid lightweight convolutions and spatial pyramid dilated convolutions achieved accurate segmentation while lowering model parameters and computations, indicating their suitability for edge applications. The proposed models demonstrated significantly higher accuracy for seedling detection and row segmentation across rice varieties with different densities, reflecting improved robustness. Their lower GPU memory usage, model complexity and faster inference speeds signify higher computational efficiency. In particular, the fast speed of the LW-Unet model indicates potential for real-time applications to facilitate timely decision-making in precision agriculture. The findings suggest that lightweight DL models can tackle complex agricultural problems with high accuracy and robustness by fusing multiscale information and adapting to varying densities. While standard deep models perform well on single tasks, lightweight models trained with limited data exhibit more generalizable capabilities for multiple similar tasks. For complex yet nuanced applications such as precision farming, more efficient and robust network architectures are instrumental for bringing insights from computer vision to the agricultural domain.

Several avenues remain for future work: first, integrating the proposed models into edge devices and UAV platforms for on-site semantic segmentation and monitoring of rice fields; second, optimizing the models for embedded devices to deploy in broader precision agriculture scenarios; and third, exploring transfer learning techniques to improve the sample efficiency of lightweight deep models for agricultural tasks. Addressing these opportunities could further unlock the potential of lightweight DL to enable data-driven decision-making and advance sustainable farming practices.

## Data Availability

The data used to support the findings of this study are available from the corresponding author upon request.
