# Supplementary material for: Lightweight Deep Learning Models for High-Precision Rice Seedling Segmentation from UAV-Based Multispectral Images
Source: Plant Phenomics. 2023 Nov 30;5:0123. doi: 10.34133/plantphenomics.0123 (PMC10688663; doi:10.34133/plantphenomics.0123)
Supplement: Supplementary 1 — Fig. S1 Tables S1 to S3 [file plantphenomics.0123.f1.zip › Supplementary materials.docx]

**Supplementary materials**


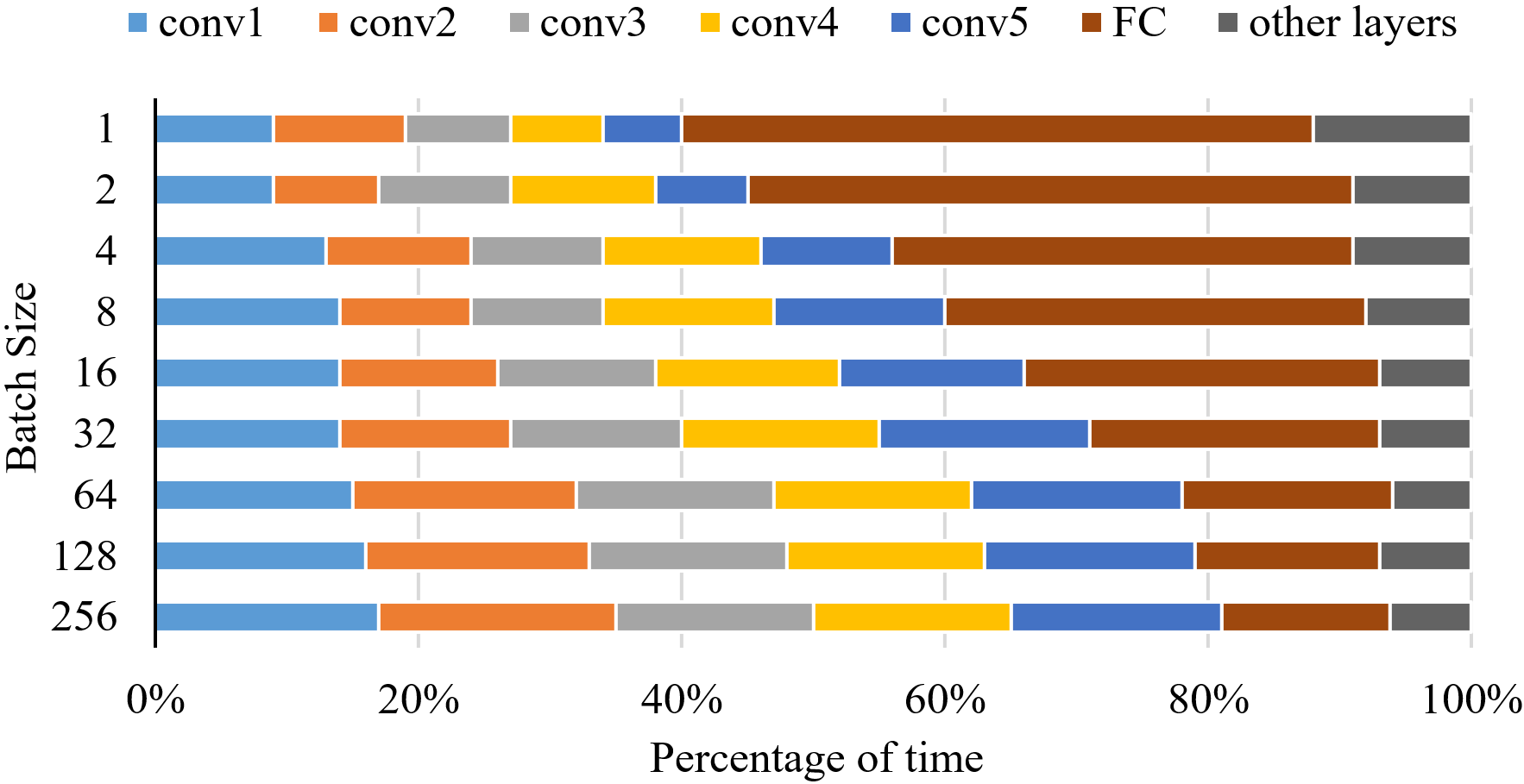


a. Computation time distribution under different batch size


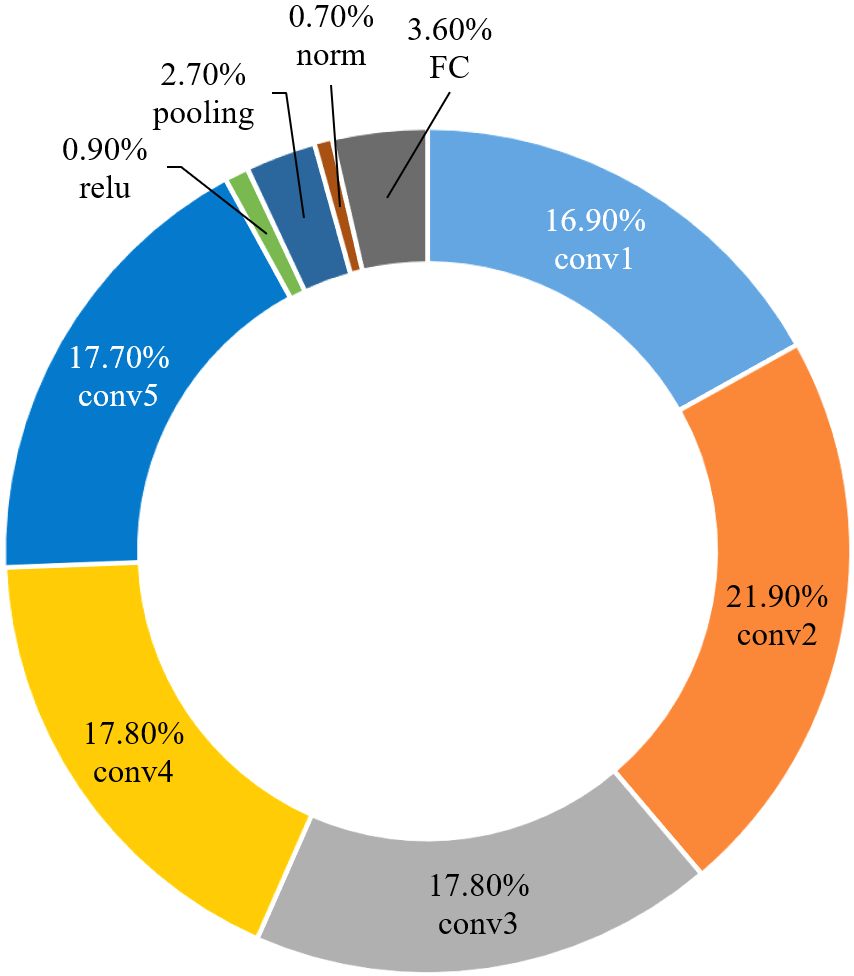


b. Model forward time distribution

**Table 1S The central wavelength and the reflectance of calibrated panel**

| Spectral band | Center wavelength/nm | Bandwidth/nm | Calibrated Reflectance panel #1/% | Calibrated Reflectance panel #2/% |
| --- | --- | --- | --- | --- |
| blue  green  red  red edge  nir | 450  560  650  730  840 | 16  16  16  16  26 | 51.02  51.21  50.04  50.47  50.66 | 25.61  25.09  25.33  26.04  25.75 |

**Table 2S Encoder-decoder configuration of the LW-Segnet.**

| Input: 512×512×5 |  | Output: 512×512×5 |
| --- | --- | --- |
| LHA-SSPDC: 512×512×64 |  | LHA-SSPDC: 512×512×64 |
| LHA-SSPDC: 512×512×64 |  | LHA-SSPDC: 512×512×64 |
| MaxPooling | → | Upsampling |
| LHA-SSPDC: 256×256×128 |  | LHA-SSPDC: 256×256×128 |
| MaxPooling | → | Upsampling |
| LHA-SSPDC: 128×128×128 |  | LHA-SSPDC: 128×128×128 |
| MaxPooling | → | Upsampling |
| LHA-SSPDC: 64×64×256 |  | LHA-SSPDC: 64×64×256 |
| LHA-SSPDC: 64×64×256 |  | LHA-SSPDC: 64×64×256 |
| LHA-SSPDC: 64×64×256 |  | LHA-SSPDC: 64×64×256 |
| LHA-SSPDC: 64×64×256 |  | LHA-SSPDC: 64×64×256 |
| LHA-SSPDC: 64×64×256 |  | LHA-SSPDC: 64×64×256 |
| MaxPooling | → | Upsampling |

Note: The left and right columns correspond to the encoder and decoder respectively. The encoding convolutional layers progress downwards, and the decoding convolutional layers progress upwards. Each conv (or deconv) layer is followed by a BN and RELU layer (not shown). The arrows indicates the pooling indices.

**Table 3S Encoder-decoder configuration of the LW-Unet.**

| Input: 512×512×5 |  | Output: 512×512×5 |
| --- | --- | --- |
| LHA-SSPDC: 512×512×64 |  | LHA-SSPDC: 512×512×64 |
| LHA-SSPDC: 512×512×64 | **⊕** | LHA-SSPDC: 512×512×64 |
| MaxPooling |  | Upsampling |
| LHA-SSPDC: 256×256×128 |  | LHA-SSPDC: 256×256×128 |
| LHA-SSPDC: 256×256×128 | **⊕** | LHA-SSPDC: 256×256×128 |
| MaxPooling |  | Upsampling |
| LHA-SSPDC: 128×128×256 |  | LHA-SSPDC: 128×128×256 |
| LHA-SSPDC: 128×128×256 | **⊕** | LHA-SSPDC: 128×128×256 |
| MaxPooling |  | Upsampling |
| LHA-SSPDC: 64×64×512 |  | LHA-SSPDC: 64×64×512 |
| LHA-SSPDC: 64×64×512 | **⊕** | LHA-SSPDC: 64×64×512 |
| MaxPooling |  | Upsampling |
| LHA-SSPDC: 64×64×1024 |  | LHA-SSPDC: 64×64×1024 |

Note: The left and right columns correspond to the encoder and decoder respectively. The encoding convolutional layers progress downwards, and the decoding convolutional layers progress upwards. Each conv layer is followed by a BN and RELU layer (not shown). The **⊕** indicates the concat process.
