# Supplementary figures and images for: Lightweight Deep Learning Models for High-Precision Rice Seedling Segmentation from UAV-Based Multispectral Images
Source: Plant Phenomics. 2023 Nov 30;5:0123. doi: 10.34133/plantphenomics.0123 (PMC10688663; doi:10.34133/plantphenomics.0123)

conv1 conv2 conv3 conv4 conv5 FC other layers

Batch Size

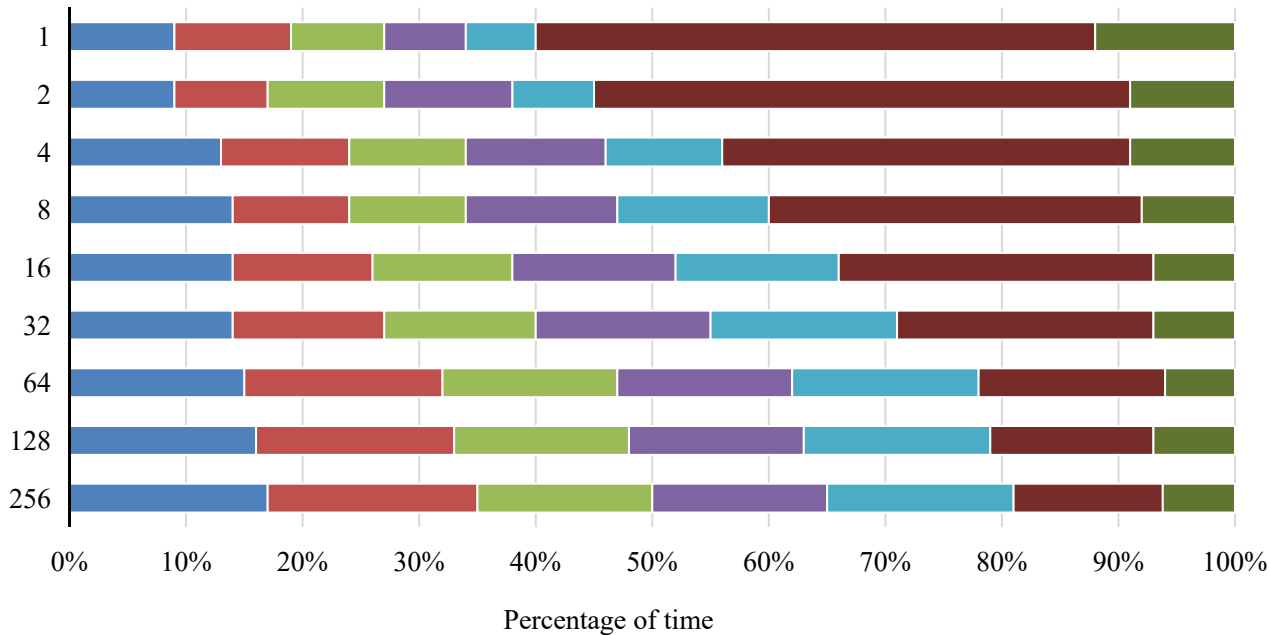

Supplement: Supplementary 1 — Fig. S1 Tables S1 to S3 [file plantphenomics.0123.f1.zip › figure1s(a).pdf]

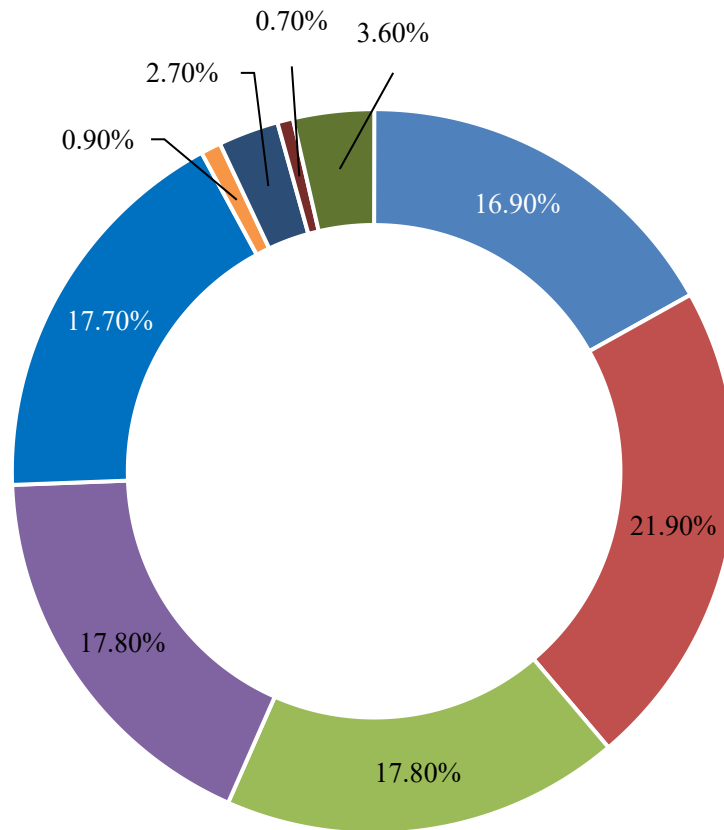

■ conv1 ■ conv2 ■ conv3 ■ conv4 ■ conv5 ■ relu ■ pooling ■ norm ■ FC

Supplement: Supplementary 1 — Fig. S1 Tables S1 to S3 [file plantphenomics.0123.f1.zip › figure1s(b).pdf]
